# Elucidation of Hosts, Native Distribution, and Habitat of the Coffee Berry Borer (*Hypothenemus hampei*) Using Herbaria and Other Museum Collections

**DOI:** 10.3389/fpls.2019.01188

**Published:** 2019-10-01

**Authors:** Fernando E. Vega, Lucy T. Smith, Nina M. J. Davies, Justin Moat, Tomasz Góral, Robert O’Sullivan, Aaron P. Davis

**Affiliations:** ^1^Sustainable Perennial Crops Laboratory, United States Department of Agriculture, Agricultural Research Service, Beltsville, MD, United States; ^2^Natural Capital Department (APD), Identification and Naming Department (LTS and NMJD), Royal Botanic Gardens, Kew, Richmond, United Kingdom; ^3^Zespół ds. Infrastruktury, Centrum Nowych Technologii Uniwersytetu, Warszawa, Poland; ^4^The Department of Life Sciences, The Natural History Museum, London, United Kingdom; ^5^School of Biological and Chemical Sciences, Queen Mary University of London, London, United Kingdom

**Keywords:** Africa, Arabica coffee, broca del café, coffee, coffee berry borer, herbaria, museum collections, robusta coffee

## Abstract

The coffee berry borer (*Hypothenemus hampei*) is the most damaging insect pest of global coffee production. Despite its importance, our knowledge on the insect’s natural habitat, range, and wild host species remains poorly known. Using archival sources (mainly herbaria but also other museum collections), we surveyed 18,667 predominantly wild-collected herbarium specimens mostly from Africa, Madagascar, and Asia for coffee berry borer occurrence. A total of 72 incidences were confirmed for presence of the coffee berry borer, with identifications assisted by micro-CT for SEM. Of the 72 positive infestations, all were from tropical African coffee (*Coffea*) species, of which 32 were from wild (non-cultivated) plants. Of the 32 wild occurrences, 30 were found in *C. canephora* (robusta coffee), 1 in *C. liberica* (Liberica coffee), and 1 in *C. arabica* (Arabica coffee). Our herbarium survey confirms literature and anecdotal reports that the coffee berry borer is indigenous to tropical Africa, and that coffee species, and particularly robusta coffee, are important hosts. We identify the wetter type of Guineo-Congolian forest as either the preferred or exclusive native habitat of the coffee berry borer. Other than coffee, we find no evidence of other naturally occurring hosts. Characters of infestation (e.g., hole position on coffee fruits) infers a certain degree of specificity between the coffee berry borer and its host.

## Introduction

The coffee berry borer [*Hypothenemus hampei* (Ferrari); Coleoptera: Curculionidae: Scolytinae] is the most damaging insect pest of coffee worldwide (Vega et al., 2015). Adult females (ca. 2 mm long) bore into coffee berries and deposit eggs within galleries in the endosperm. The adults and their progeny feed on the coffee seeds, greatly reducing the quality and yield of the marketable product. In this way, the coffee berry borer causes an estimated US$215–358 million in yearly losses in Brazil (Oliveira et al., 2013). Based on this estimate, it is very likely that worldwide losses are over US$500 million, considering that there are 82 coffee-producing countries worldwide (FAOSTAT, 2019) and all the largest producers have reported the presence of the insect (Vega et al., 2015). Our knowledge of the insect’s natural habitat, range, and particularly wild host species remains woefully inadequate. Effective pest management strategies against the coffee berry borer remain elusive due to the insects’ cryptic life habit; therefore, a better understanding of host and host specificity could be instrumental in finding alternative, more effective means for managing the insect.

The coffee berry borer is considered to be a monophagous species, i.e., it is believed to feed solely on coffee seeds inside the coffee berry (Vega et al., 2015). There are 124 coffee (*Coffea*) species, which occur naturally in the tropical Old World, of which 47 species occur in tropical Africa (Davis et al., 2006; Davis et al., 2019). Of these species, it has been suggested (Baker, 1984; Damon, 2000; Jaramillo et al., 2009; Jaramillo et al., 2011), but not demonstrated, that robusta coffee (*Coffea canephora*), a species naturally occurring at lower altitudes (250–1500 masl) in tropical Africa (Davis et al., 2006), might be the original host of the coffee berry borer. These suggestions are most likely based on literature records in which Uganda features prominently. For example, Thomas (1944) states that the coffee berry borer is common on wild *C. canephora* in the forests of Uganda, while it has also been stated that in Uganda the host plants for the insect are *Coffea* species, including *C. canephora* (Hargreaves, 1926; Thomas, 1940). Referring to the insect, Thomas (1940) states that “it is indigenous here (as well as other tropical African countries) and has been found in all our coffee areas [Uganda] south of 2° N. latitude.” In his discussion concerning *C. canephora* cultivation in Bukoba (NW Tanzania), Jervis (1939) states that “[*Hypothenemus hampei*] is no less indigenous to East Central Africa than the coffee tree itself.”

According to Beille (1925), the destruction of African forests led to the coffee berry borer moving from forest habitats into Arabican (*C. arabica*), robusta, and Liberica (*C. liberica*) plantations. This transition to cultivated coffee is important because, as mentioned above, many coffee species are endemic to tropical African forests, but the number of coffee plants drastically increased as coffee production intensified and expanded across Africa, particularly since the beginning of the 20^th^ century, providing the insect with ample resources. Despite the focus on *Coffea* as the host, other (non-*Coffea*) fruit-producing trees of tropical Africa have also been identified as potential hosts of the coffee berry borer (Vega et al., 2012). Schedl (1960) reported the presence of the coffee berry borer in 20 plant genera belonging to 13 families in the Democratic Republic of Congo (Vega et al., 2012) and stated (translated from French; see Vega et al., 2012): “It is interesting, from the biological point of view, to note that after the investigations conducted by the author in Yangambi and after the bibliographical data on the subject of *Stephanoderes* [*Hypothenemus*] *hampei* Ferr. there exists inside the rainforest a series of natural hosts for the parasite that give it the possibility to develop independently from coffee plantations.” Away from Africa, and on the basis of molecular data, Asia, and specifically Java, has been posited as a separate origin for coffee berry borer and its hosts (Gauthier, 2010).

To test the ideas of Beille (1925), reports of Schedl (1960), the notion that the coffee berry borer is monophagous, and that *C. canephora* is a natural host (Baker, 1984; Damon, 2000; Jaramillo et al., 2009; Jaramillo et al., 2011), it would be necessary to undertake time-consuming and expensive fieldwork across tropical Africa and Asia. Therefore, we decided to conduct a large-scale survey of herbarium specimens (with support from other museum collections) for the occurrence of possible hosts, including coffee and other plants as reported by Schedl (1960) and listed in Vega et al. (2012). The use of herbarium specimens to circumvent what might be impractical fieldwork or to have broader field coverage has been discussed by Beaulieu et al. (2017) and Meineke et al. (2018).

Using herbarium specimens for purposes beyond plants, and more specifically, to study plant–herbivore interactions, has been reported by others. For example, Beaulieu et al. (2017) used herbarium specimens to assess insect pressure on the invasive weed purple loosestrife (*Lythrum salicaria*), from the invasion phase to the saturation phase. Meineke et al. (2018) compared insect herbivory on four plant species in herbarium specimens collected over 112 years (i.e., between 1896 and 2008), and found a significant increase in herbivory in specimens from the 2000s when compared to specimens from the 1800s. Working with two bamboo species, Stern (1993) sampled stem galls in herbarium specimens and dissected the gall-forming midges (Diptera: Cecidomyiidae) as well as the hymenopteran parasitoids that attack the midges, thus revealing previously unrecorded plant hosts. Abbott et al. (1999) examined more than 500 specimens of two *Eucalyptus* species in five herbaria to assess the historical incidence of two leaf-miner species (Lepidoptera: Incurvariidae). Also working with *Eucalyptus*, Morrow and Fox (1989) sampled herbarium specimens to assess insect damage prior to modern disturbances of the environment. Webber et al. (2007) sampled specimens from 15 herbaria to assess ant–plant associations in 27 species of *Ryparosa*, an understory rainforest tree. The herbarium specimens greatly expanded the knowledge on ant–plant traits associations (i.e., myrmecotrophic, myrmecophytic, and myrmecoxenic plant species). Lees et al. (2011) examined specimens from six herbaria to determine the geographic range of a highly invasive leaf-mining moth (Lepidoptera: Gracillariidae). In an interesting twist, they extracted DNA from larvae and pupae extracted from the leaf mines in the archival samples and confirmed the identity of the moth and its presence in the Balkans in 1879. Finally, Veenstra (2012) examined Australian herbarium specimens of *Leptospermum laevigatum*, a native shrub now considered to be a weed, with the goal of determining the geographic distribution and historical incidence of a gall midge that attacks the shrub, as well as of a parasitoid wasp that attacks the midge. The results expanded on the distribution of the gall midge, and an herbarium specimen collected in 1875 revealed incidence of the gall midge as well as the parasitoid.

The specific questions for our herbarium survey were: (1) Would examination of targeted taxa including *Coffea* and other plant groups (Schedl, 1960; Vega et al., 2012) stored in herbarium collections reveal coffee berry borer incidence? (2) If we detected the coffee berry borer, could these data be used to confirm or refute reports of host status? (3) Are there other, as yet unreported, alternative hosts of the coffee berry borer for plant species related to coffee (tribe Coffeeae, family Rubiaceae; Davis et al., 2007; Tosh et al., 2009; Arriola et al., 2018; Cheek et al., 2018).

To answer these questions, we examined 18,667 herbarium specimens from several herbaria. All insect infestations were recorded and then examined further for the presence (positive identification) of the coffee berry borer. Identifications were assisted by using light microscopy and micro-computed tomography for scanning electron microscopy (micro-CT for SEM), the latter to also image insects on and within herbarium fruits. In the absence of the insect, analyses were undertaken to link coffee berry borer infestation to characteristics of the hole bored into fruits (diameter, shape, and position on the fruit). The natural (wild) distributions of host plants and the coffee berry borer were calculated using the range metric extent of occurrence (EOO) to examine distribution overlap between host species and the coffee berry borer and to determine their natural habitat.

## Materials and Methods

### Herbaria Visited and Plant Material (Database 1)

Herbarium surveys were conducted at herbaria holding large collections of Old World (and especially African) specimens, including Royal Botanic Gardens, Kew (K); Natural History Museum, London (NHM); British Museum, London (BM); Muséum National d’Histoire Naturelle, Paris (P); National Botanic Garden of Belgium, Meise (BR); and National Herbarium Nederland, Wageningen (WAG). Herbarium abbreviations follow Holmgren et al. (1990). We sampled the holdings of these collections for all African, Madagascan and Asian *Coffea* species (Davis et al., 2006; Davis et al., 2019), species of African tribe Coffeeae, and other species of Rubiaceae and non-Rubiaceae reported as hosts of the coffee berry borer (Beille, 1925; Schedl, 1960; Vega et al., 2012). In total, we examined 18,667 specimens (herbarium sheets), including 7,861 for *Coffea*, 14,568 for all Coffeeae, 299 for other (non-Coffeeae) Rubiaceae, and 3,800 for other plant families (**Table 1**). In our database we recorded: family, genus, country, and area [using the Taxonomic Databases Working Group (TDWG) geographical scheme] (Brummitt et al., 2001), at levels 1 (continent), 2 (region), and 3 (botanical country—where most regions are subdivided into units generally equating to a political country, but large countries may be split or outlying areas omitted), whether cultivated or wild, herbarium consulted, number of specimens, number fruiting, all potential beetle infestations, and notes. A summary of the families, genera, and species examined is given in **Table 1**. It is important to make clear the difference between the number of collections and the number of specimens. A collection is the result of a single collection event, and represented by a unique identifier (collectors name and number; or if the collection number is lacking collectors name and date), as opposed to a (herbarium) specimens (or sheet), which may represent more than one sheet (duplicate) of each collection.

**Table 1:** Summary of taxa examined for herbarium coffee berry borer survey, with number (and %) of specimens and number (and %) of fruiting specimens examined, and number of specimens with coffee berry present.

Family	Genus	Species	Natural distribution	Herbarium specimens (all)	Herbarium specimens (fruiting only)	Herbarium specimens % fruiting	Number and % of fruiting specimens with CBB
Achariaceae	*Caloncoba*	*crepiniana*	Central Africa	106	27	25.47%	0
Achariaceae	*Caloncoba*	*glauca*	West & Central Africa	45	16	35.56%	0
Apocynaceae	*Pleiocarpa*	*pycnantha*	Tropical Africa	504	120	23.81%	0
Bigoniaceae	*Spathodea*	*campanulata*	Tropical Africa	454	62	13.66%	0
Calophyllaceae	*Mammea*	*africana*	West & Central Africa	315	63	20.00%	0
Clusiacease	*Allanblackia*	*floribunda*	West Africa	606	63	10.40%	0
Combretaceae	*Terminalia*	*superba*	Tropical Africa	157	50	31.85%	0
Leguminosae	*Caesalpinia*	*pulcherrima*	Tropical Africa	113	43	38.05%	0
Leguminosae	*Cathormium*	*altissimum*	West & Central Africa	269	131	48.70%	0
Leguminosae	*Dialium*	*englerianum*	West & Central Africa	155	81	52.26%	0
Leguminosae	*Prioria*	*oxyphylla*	West & Central Africa	171	66	38.60%	0
Malvaceae	*Cola*	*grisaefolia*	West & Central Africa	43	2	4.65%	0
Meliaceae	*Trichilia*	*gilgiana*	West & Central Africa	208	67	32.21%	0
Myristicaceae	*Pycnanthus*	*angolensis*	West & Central Africa	452	111	24.56%	0
Rhizophoraceae	*Anopyxsis*	*klaineana*	West & Central Africa	202	70	34.65%	0
**Subtotal (non- Rubiaceae)**				**3,800**	**972**	**25.58**%	**0**
*Coffeeae*							
Rubiaceae	*Argocoffeopsis*	(Numerous spp.)	West & Central Africa	885	234	26.44%	0
Rubiaceae	*Belonophora*	(Numerous spp.)	West & Central Africa	357	152	42.58%	0
Rubiaceae	*Calycosiphonia*	(Numerous spp.)	Tropical Africa	329	125	37.99%	0
Rubiaceae	*Coffea*	43 species*	Tropical Africa	6,059	2,725	44.97%	72 (2.56%)
Rubiaceae	*Coffea*	53 species	Madagascar & Mascarenes	1,700	617	36.29%	0
	*Coffea*	7	Asia and Australasia	102	42	41.18%	0
Rubiaceae	*Empogona*	Numerous spp.	West & Central Africa	284	93	32.75%	0
Rubiaceae	Oxyanthus*	Numerous spp.	Tropical Africa	2,878	1,143	39.72%	0
Rubiaceae	*Sericanthe*	Numerous spp.	West & Central Africa	258	127	49.22%	0
Rubiaceae	*Tricalysia*	Numerous spp.	Tropical Africa	1,716	416	24.24%	0
**Subtotal (Rubiaceae**—**Coffeeae)**				**14,568**	**5,674**	**38.95%**	**72 (1.23%)**
*Non-Coffeeae*							
Rubiaceae	Nauclea*	*diderrichii*	West & Central Africa	293	138	47.10%	0
Rubiaceae	*Nostolachma*	*khasiana*	India	6	4	66.67%	0
**Subtotal (Rubiaceae**—**Non-Coffeeae)**				**299**	**142**	**47.49%**	**0**
**Total (Rubiaceae)**				**14,867**	**5,816**	**39.12%**	**72 (1.20%)**
**Totals**				**18,667**	**6,788**	**36.36%**	**72 (1.03%)**

CBB, coffee berry borer. All Rubiaceae genera are members of tribe Coffeeae, apart from Oxyanthus (Gardenieae) and Nauclea (Naucleeae). Numbers are for specimens (sheets): there may be more than one specimen (i.e., a duplicate) per collection (see **Table 2** for collections). In the case of the 72 coffee specimens, the number of collections is the same (i.e., one specimen (sheet) per collection). * Includes seven unnamed taxa and unidentified specimens.

### Insect Reference Material

We examined the coffee berry borer collection at NHM, recording data provided on the labels (including origin and hosts) and associated material (some specimens were on coffee seeds). A total of 96 specimens were examined. Representative material was imaged using micro-CT for SEM (see below) for comparison with the beetles found inside coffee fruits from the various herbaria and for identification purposes (as standard reference material).

### Recording Beetle Infestations (Database 2)

We recorded all potential beetle infestations of fruits, including those that were clearly not the coffee berry borer. The same information was captured as for Database 1, with the addition of the plant collectors name and number, date (year only), micro-CT for SEM details, herbarium of specimen origin, and basic details of infestation (hole size, shape, and position), and potential coffee borer presence. A total of 135 collections were entered into the database.

### Insect and Coffee Berry Borer Identification

Specimens recorded in Database 2 were examined for positive occurrence of the coffee berry borer. A key criterion used for identifying the insect is size (ca. 2 mm long × 0.6 mm wide for females) and the shape of the interstrial setae. The latter is more slender and cylindrical than the broader and flattened interstrial setae in other *Hypothenemus* species (*H. crudiae*, *H. eruditus*, *H. obscurus*, *H. seriatus*) that have been collected (but cannot reproduce) inside side coffee berries (Fonseca, 1937; Garcia Martell, 1972; Wood, 1982; Vázquez et al., 1996; Wood, 2007; Constantino et al., 2011); see Atkinson (2019) for photos. We used micro-CT for SEM (see below) to image potential coffee berry borers, for identification using key morphological characters and *via* comparison with standard specimens identified by beetle taxonomists and held at NHM (see above). We also recorded the position, shape, and diameter of the entrance/exit holes in fruits, in all cases, i.e., where a beetle was evident (entering or exiting the fruit) or not seen (already inside the fruit, or where the beetles had vacated the fruit), and the ratio of fruits infested. The position on the fruit was recorded at ‘apical’ [subdivided into: (1) within the floral disc (ID), i.e., the small (2–10 mm) disc-shaped protuberance at the apex of the fruit; (2) touching the floral disc (TD); and near the disc (ND), in the upper 1/3 of the fruit body]; ‘side’ (SD), the middle 1/3 of the fruit; and ‘basal’ [subdivided into: touching the fruiting stalk (pedicel) (TP); and near the pedicel (NP) in the lower 1/3 of the fruit]. The hole bored by the coffee berry borer is reported as being ‘perfectly round’ (Fonseca, 1937). The diameter was recorded using either a binocular microscope (Leica MZ9.5, Leica Microsystems, Wetzlar, Germany) or a graticuled hand-lens (Leitz, 8X, with 0.1 mm divisions; Leitz, Wetzlar, Germany).

### Recording Coffee Berry Borer Infestations (Database 3)

All positive coffee berry borer identifications (i.e., beetle positively identified), or tell-tale/characteristics of coffee berry infestation (hole position, shape, and diameter; see above), and ratio of fruits infested (per specimen) were recorded into Database 3. We also recorded the same information as collected in Database 2, but the data was noted for each fruit (some collections have multiple sheets per accession, i.e., for each unique identifier). A total of 363 fruits (70 specimens) were entered into the database.

### Light Microscopy and Micro-CT for SEM

All insect occurrences were initially examined using a binocular microscope (Leica MZ9.5, Leica Microsystems, Wetzlar, Germany) for initial identification. To enable precise identification of the coffee berry borer, and other beetles, micro-CT for SEM imaging was undertaken as it is a non-destructive technique, which captures the micromorphology of a specimen using X-ray micro-focused computer tomography. Uncoated samples were placed under low vacuum conditions in a LEO 1455VP scanning electron microscope (Carl Zeiss Microscopy GmbH, Göttingen, Germany). Electron micrographs were acquired with a four-quadrant backscattered electron detector (K.E. Developments Ltd, Cambridge, England) in a TOPO mode to enhance surface topography. Images were recorded with the following microscope setup: chamber pressure = 20 Pa; acceleration voltage = 20 kV; working distance = 15–18 mm; pixel size = 115 nm to 4.1 µm.

### Calculation of Extent of Occurrence and Broad-Scale Environmental Niche for Coffee Species and the Coffee Berry Borer

Cleaned and verified, georeferenced distribution (point) data for naturally occurring (wild) African coffee species (Davis et al., 2019; Moat et al., 2019), and coffee berry borer occurrences based on the results of our herbarium survey, were used to calculate the EOO of each species. To calculate the EOO areas, the point data was projected to the Africa Albers Equal Area Conic projection (central meridian 25 degrees and standard parallels -23 and 20 degrees) in R (R Core Team, 2016) using the rgdal package (Bivand et al., 2019). EOO polygons were calculated using a convex hull (the smallest polygon encompassing all points) using the R packages rCAT (Moat, 2017) and sp (Pebesma and Bivand, 2005). The EOOs for each African coffee species and coffee berry borer occurrence were mapped, and the percentage overlap was calculated using the Rpackage raster (Hijmans, 2016) with the command ‘intersect’ to produce a 44 × 44 matrix (**Supplementary Material Table S1**). Coffee berry borer incidence and coffee species overlapping with the distribution of the insect were allocated to a corresponding major vegetation type, and with reference to coffee species habitat summaries (Davis et al., 2006), a coffee specimen database (RBG Kew), and *via* the inspection of satellite imagery with our datapoints mapped onto Google Earth (Google Earth, 2019).

## Results

### Incidence of the Coffee Berry Borer From the Large-Scale Herbarium Survey

Examination of fruiting material was key to this study, as this is the only stage on which the coffee berry borer can be detected. Of the 18,667 specimens (herbarium sheets) examined (**Table 1**), 6,788 had fruits present (36.4%) excluding the very early stages of fruit production; the remainder were either flowering or sterile (no flowers or fruits). Various insect infestations were found in 135 collections (data not shown), with coffee berry borer confirmed in the fruits of 72 coffee specimens (= 72 collections) (**Tables 1** and **2**).

**Table 2:** Results of survey for African coffee species, with number of collections, and number and percentage of collections with coffee berry borer, disaggregated according to whether cultivated/farmed (cult.) or of wild origin. All figures refer to fruiting collections only.

Genus	Species	Number of collections	Number of cult. collections	Number of wild collections	Number of cult. collections with CBB	Number of wild collections with CBB	Total Number of collections with CBB (cult. and wild)	% of cult. (against total number) of collections with CBB	% of wild collections with CBB, against total number of all cult. collections	% of wild collections with CBB, against total number of all wild collections
*Coffea*	*Arabica*	211	175	36	6	0	6	100.00%	0.00%	0.00%
*Coffea*	canephora*	285	136	149	22	30	52	42.31%	57.69%	20.13%
*Coffea*	*Congensis*	54	16	38	2	0	2	100.00%	0.00%	0.00%
*Coffea*	*Liberica*	279	132	147	5	1	6	83.33%	16.67%	0.68%
*Coffea*	*Stenophylla*	13	6	7	1	0	1	100.00%	0.00%	0.00%
*Coffea*	*mayombensis*	82	1	81	0	1	1	0.00%	100.00%	1.23%
	Totals*	**924**	**466**	**458**	**36**	**32**	**68**	**52.94%**	**47.06%**	**6.99%**

CBB, coffee berry borer. A collection represents a unique collection event (the collectors name and number (or date) representing a unique identifier). Collections may be represented by one or more specimens (sheets); see **Table 1** for specimen (sheet) numbers. * Four coffee berry borer records are not included in this list: there was no data for two C. canephora specimens regarding cultivated (cult.) vs. wild status and two specimens were cultivated specimens from Asia (the hybrid Coffea × crameri and C. canephora).

### Identification of the Insect and Hosts From Herbarium Material

**Figure 1:**
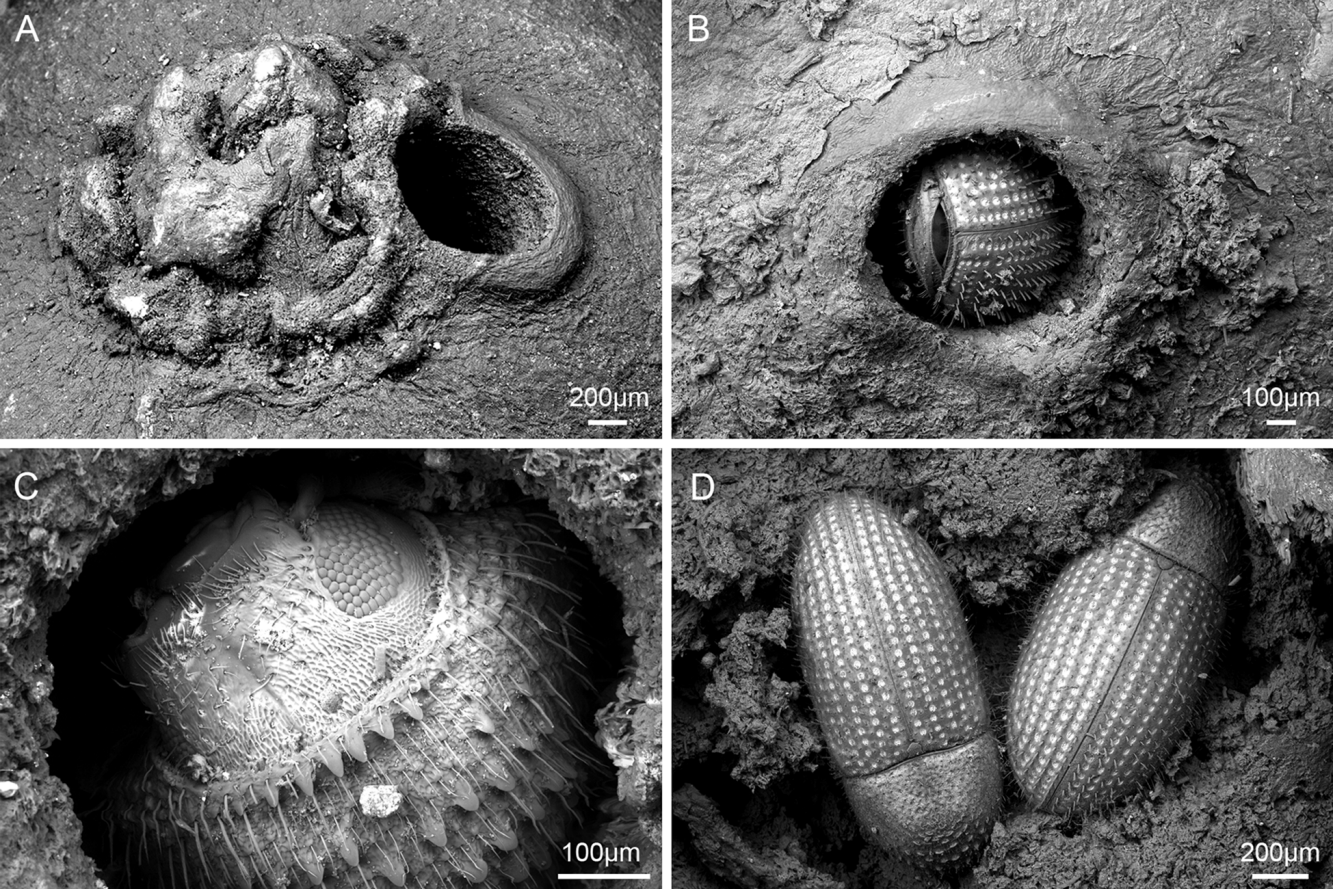
Coffee berry infestation in *C. canephora*. **(A)** Apex of fruit, with entry hole next to the disc (nectary) made by the coffee berry borer; specimen Soors b178 [BR], Democratic Republic of Congo, cultivated, 1934. **(B)** Apex of fruit, entry through the disc; specimen Myers 10212 [K], Democratic Republic of Congo, wild collected, 1938. **(C)** Apex of fruit, exit adjacent to disc, close-up of coffee berry borer head; specimen Soors b178 [BR], Democratic Republic of Congo, cultivated, 1934. **(D)** Endosperm, fruit, and seed previously opened, showing two of seven coffee berry borers in a single seed; specimen Myers 1631 [K], Cameroon, wild collected, 1987.

**Figure 2:**
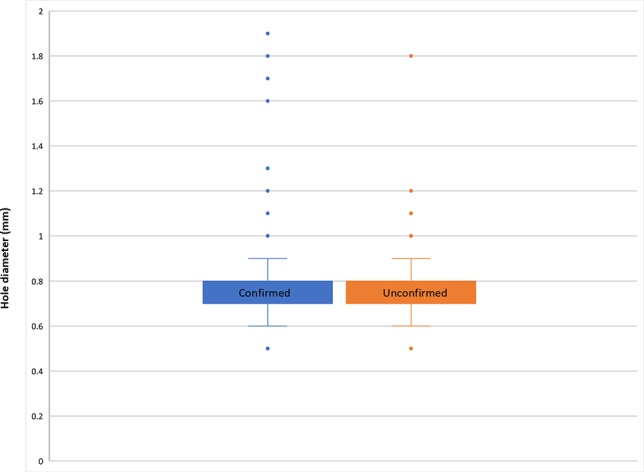
Box and whisker plot of hole diameter in coffee fruits, for both confirmed (blue; positive identification for coffee berry borer, with insect in situ) and non-confirmed (orange; coffee berry borer beetle infestation assumed by beetle not visible, i.e., hidden within the fruit), based on measurement of 545 holes (363 fruits) from 36 coffee collections (see Results section Identification of the Insect and Hosts from Herbarium Material).

Using a combination of light microscopy and micro-CT for SEM, potential coffee berry borer occurrences were found on 37 collections (37 specimens): 36 were confirmed as coffee berry borer (all restricted to *Coffea* species), and 1 as an unidentified *Hypothenemus* species (i.e., not *H. hampei*), found on *Mammeaafricana* (Calophyllaceae). Therefore, there were 36 collections with coffee berry borer beetles *in situ*, all restricted to *Coffea*. The coffee berry borers were found either entering (**Figure 1B**) or exiting (**Figure 1D**) the coffee fruit or, in the case where fractured seeds were available, within the main tissue (endosperm) of the seed (**Figure 1D**). The 36 collections (36 specimens) had the distinctive small (ca. 0.8 mm), round hole (e.g., **Figures 1A–C**), indicative of the infestation, but no insect visible (as it is probably inside the fruit). We measured 545 holes from 363 fruits, from 36 fruiting coffee collections. For those with confirmed coffee berry borer (i.e., insect positively identified), the mean hole diameter was 0.78 mm (min. 0.5 mm, max. 1.9 mm) and for unconfirmed coffee berry borers (holes present but no beetles) the mean was 0.8 mm (min. 0.5 mm, max. 1.8 mm). The box and whisker plots (**Figure 2**) show the remarkable uniformity of hole diameter (**Figures 1A–C**). The coffee berry borer beetles examined during this study had a width of 0.6–0.7 mm. In a few cases, we examined two beetles in a single hole, with a hole diameter of 1.8 mm; this explains the rare occurrence of the larger holes (**Figure 2**). In the unconfirmed cases (i.e., specimen lacking the insect), additional holes of regular size were also present, if not on the same fruit, the fruit belonging to the same collection. Fruit tissue shrinkage (upon drying) may explain the smaller holes found in some specimens.

We measured the position of 501 holes, from 36 coffee collections; for those with confirmed coffee berry borers (i.e., insect present and positively identified), 71.9% were from the apical portion (ID = 17.5%, TD = 49.4%, ND = 5%), 7.8% from the side (SD), and 20.3% from the basal portion (TP = 8.5%, NP = 11.8%); for those with unconfirmed coffee berry borers (beetle not present, or hidden inside the fruit), 79.4% were from the apical portion (ID = 28.4%, TD = 41.2%, ND = 9.8%), 11.8% from the side (SD), and 8.8% from the basal portion (TP = 2.9%, NP = 5.9%). The percentage of holes in the apical portion was similar for both confirmed (71.9%) and unconfirmed (79.4%), as was the percentage of holes touching the disc (49.4% vs. 41.2%), although the percentages for side and basal holes varied between the two categories (7.8% vs. 11.76%, and 20.3% vs. 8.8%, respectively). All of the holes were perfectly round; the coffee berry borer appears to cut into the fruit by moving its body through a circular eating-movement motion path.

From the data on hole size, shape, and position we concluded that the combination of hole size, shape, and position was unique to the coffee berry borer and added the 36 ‘possible’ coffee berry borer beetle infestations to the total for positive occurrence (i.e., 72 in total).

### Review of the Coffee Berry Borer Collections at the NHM London

Of the 96 coffee berry borer collections examined at the NHM, 17 were recorded with host information (two with the coffee seeds also collected), with a further four not recorded for the host but with the host collected as part of the specimen (i.e., 21 records in total). Sixteen were recorded as coffee being the host (species not given; although two had the seeds present as part of the collection: one was *C. canephora* and one *C. liberica*); of the four where the seeds were present (but where there was no written record), two were *C. canephora*, and two were *C. liberica*. Of the 20 coffee records, 12 were from Africa and 4 were from Asia; there are no indications whether the African records were from wild or cultivated sources; cultivated coffee (*C. arabica*, *C. canephora* and *C. liberica*) is introduced in Asia. The only non-coffee record host report is for *Caesalpinia pulcherrima* (Leguminosae) from Lusambo in the Democratic Republic of Congo, although the host plant material was not present.

### Features of Coffee Berry Borer Infestation in African Coffee Specimens

Of the 6,059 African coffee specimens examined (for 43 species across Tropical Africa; **Table 1**), 2,725 had fruits present (44.9%), with 72 specimens (and collections) (2.6%) having coffee berry borer infestation. Of the coffee species surveyed, only six species were found to be infested (**Table 2**). The total number of recorded infestations per collection (fruiting material) is low in all species except *C. canephora* (at 18.3%); in the other species, the rates range from 1.2 to 7.7% (**Table 2**). Disaggregation on the basis of whether the collections were collected from cultivated or wild sources gives the following results: *C. arabica* 100% (cultivated) vs. 0% (wild); *C. canephora* 42.3% (cultivated) vs. 57.7% (wild); *C. congensis* 100% (cultivated) vs. 0% (wild); *C. liberica* 83.3% (cultivated) vs. 16.7% (wild); *C. stenophylla* 100% (cultivated) vs. 0% (wild); *C. mayombensis* 100% (wild) vs. 0% (cultivated) (**Table 2**). *Coffea mayombensis* has a high wild infestation rate per recorded coffee berry borer incidence (100%), but overall, the infestation rate is low: i.e., 1.2% of all fruiting collections.

### Habitat and Natural Distribution of the Coffee Berry Borer and Its Hosts

**Figure 3:**
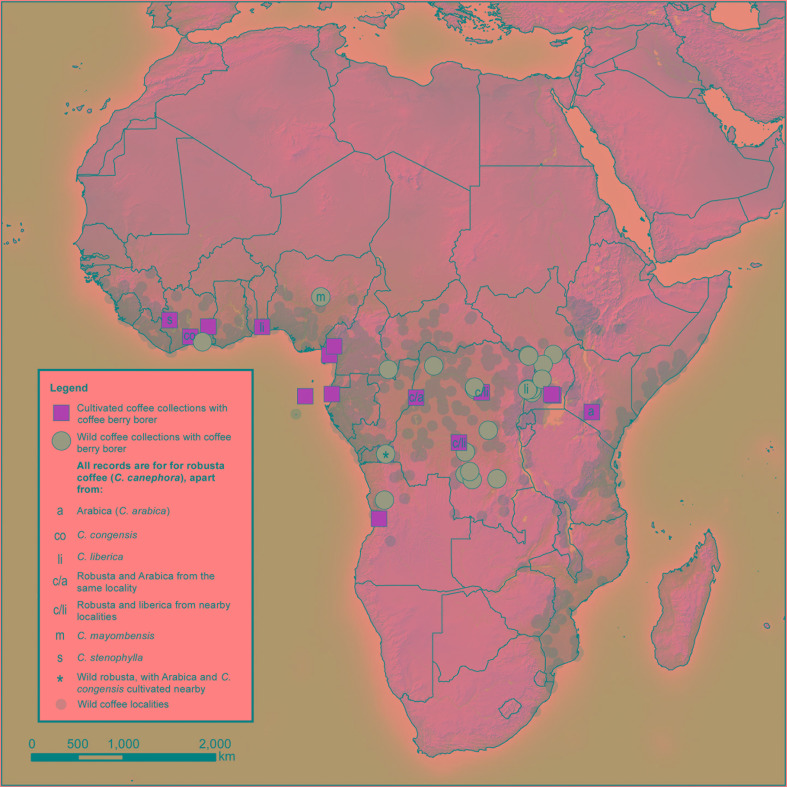
Distribution of coffee berry borer incidence based on herbarium survey. All data based on herbarium specimen records (**Table S1**). Natural distribution of all wild African coffee species (small grey dots) from Davis et al. (2019) and Moat et al. (2019).

From the distribution of our wild coffee berry borer occurrences (**Figure 3**), we deduce that the natural habitat of the insect is lowland African rainforest, or more precisely the wetter type of Guineo-Congolian forest (White, 1983). Numerous coffee species (wild occurrences) were found to have distribution areas (EOO) overlapping with the coffee berry borer and also confined to the wetter Guineo-Congolian forest type. Using the more ‘inclusive’ EOO overlap calculation [i.e., % coffee species EOO overlap with coffee berry borer EOO; first and last column (vertical axis) in **Table S2**], those with more than 30% overlap in EOO were (percentage EOO overlap in parentheses): *C. brevipes* (53%), *C. canephora* (57%), *C. liberica* (95%), *C. mannii* (83%), and *C. mayombensis* (39%). Using the ‘less inclusive’ EOO overlap calculation [% coffee berry borer EOO overlap with coffee species EOO; first and last row (horizontal axis) in **Table S2**], several further species had shared distributions. In the latter group, *C. kivuensis* (1,900–2,100 masl) and possibly some populations of *C. eugenioides* (300–2,000 masl; lower altitude localities are outside the wetter Guineo-Congolian forest type) would be excluded as their elevation occurrence places them outside the wetter type of the Guineo-Congolian forest. On inspection of collection point data viewed in Google Earth at a large scale (10 km plus), the distribution of many coffee species appears to overlap. However, on a smaller scale (<10 km), it is clear that species rarely overlap, and only very infrequently are they found in the same locations, which is supported by observation made during fieldwork for coffee in West and Central Africa (A. Davis, pers. observ.). In exceptional cases, it is possible to find species in the same forest patch; some notable examples for Guineo-Congolian species include *C. montekupensis* – *C. bakossi* – *C. brevipes* – *C. mannii* – *C. canephora* (less common) in Cameroon, and *C. canephora* – *C. liberica* in Uganda, although even here there is often niche separation (A. Davis, pers. observ.).

## Discussion

### Identification of Natural (Wild) Hosts, Distribution, and Habitat Type of the Coffee Berry Borer

On the basis of the number of infestations for individual specimens (herbarium sheets) and collections (unique collecting events, often with multiple sheets), coffee (*Coffea*) is the only genus in the Coffeeae, the Rubiaceae, and indeed of all specimens examined in this study, with records for positive coffee berry borer incidence. Of the *Coffea* species identified as hosts, only *C. canephora*, *C. liberica*, and *C. mayombensis* are confirmed with coffee berry borer infestations for wild populations; coffee berry borer infestations for *C. arabica*, *C. congensis*, and *C. stenophylla* were restricted to cultivated (farmed) collections (**Table 2**). The incidence is highest in *C. canephora*, where 30 of 149 (20.1%) wild fruiting collections were infested; in *C. liberica*, it was 1 of 147 (0.7%), and *C. mayombensis* 1 of 81 (1.23%). All three species overlap in distribution (EOO) with the coffee berry borer; under the ‘most inclusive’ EOO calculation, *C. canephora* (100%) has the highest percentage overlap, *C. liberica* (95%) the second highest, and *C. mayombensis* (39%) the fifth (out of 15 with >1% overlap) (**Table S2**). These three species share the same general habitat of the coffee berry borer, that is, African lowland rainforest, or, more precisely, the wetter type of Guineo-Congolian forest (White, 1983). This habitat corresponds well with recorded data and observations for coffee berry borer infestation, which report that the optimum conditions for the insect include high temperatures and moisture (Leroy, 1936); high rainfall of 80 inches (2030 mm), stated as “closely resembling those in its natural habitat, the rainforests of the Congo,…” and where “extremely humid conditions prevailed” (Jervis, 1939). This may explain why coffee berry borer infestations might be lower than otherwise expected in areas the beetle is known but is rarely a major pest, such as the drier, lowland areas of Uganda where *C. canephora* is cultivated in large quantities. This may also be the case for lower rainfall and lower temperature areas for *C. arabica* cultivation in Ethiopia (Davis et al., 2018), although high infestation rates in that country are reported in areas of high cultivation intensity (Mendesil et al., 2003; Mendesil et al., 2004) biasing a possible climatic signal.

Given the infestation data for the coffee berry borer and host coffee species, we therefore propose that *C. canephora*, *C. liberica*, and *C. mayombensis* are natural hosts for the coffee berry borer, and that of these, *C. canephora* is the most significant. In the case of *C. canephora*, our findings would support the observations recorded in the literature for the natural host status of *C. canephora* in western Uganda (Hargreaves, 1926; Thomas, 1940), and Bukoba in western Tanzania (Jervis, 1939), as well as literature accounts for *C. liberica* (Thomas, 1940). Thomas (1940) also reports infestation on *C. liberica* and *C. eugenioides*, both indigenous to Uganda, and although it is not stated whether he is referring to wild or cultivated plants, these species were only cultivated in very small quantities, if at all.

Given that a range of coffee species may be infested with the coffee berry borer, we have to accept the possibility that wild coffee populations could have become infested from farmed stock, as natural forest was cleared or altered for coffee cultivation. This is more likely to be the case from the early 20^th^ century onwards, when coffee cultivation increased dramatically in Africa. *Coffea canephora* was being farmed from at least the mid-19^th^ century onwards in East Africa (Wrigley, 1988), and the west coast of Africa (based on herbarium specimen records), and it is from many of these collections that the coffee berry borer was collected. The type species of *H. hampei*, described as *Cryphalus hampei* Ferrari, was found within a coffee sample from an unknown origin and imported into France (Ferrari, 1867). Herbarium records of coffee predating earliest cultivation of coffee are rare, as botanical exploration of Africa, particularly inland, did not start in earnest unto the end of the 19^th^ and early-20^th^ centuries. For the collections showing positive coffee berry borer identifications, the percentages across approximately two-decadal time intervals are 1900–1918 (34.7%), 1919–1941 (41.7%), 1945–1963 (12.5%), and 1975–1995 (11.1%). For the collections showing positive coffee berry borer identifications for wild host plants, the percentages across approximate two-decadal time intervals are 1902–1924 (15.6%), 1919–1941 (56.25%), 1946–1963 (21.88%), and 1980–1987 (6.3%). Untangling these sketchy data from collection effort is all but impossible, given the number of unknown variables, but there is correspondence between coffee berry borer incidence and the exponential growth of coffee cultivation in Africa.

It is therefore intriguing that coffee species that overlap in distribution (EOO) and habitat with our recorded occurrences of the coffee berry borer, from both cultivated and wild sources, have no evidence of infestation, including those species of coffee where we have examined a high number of fruiting specimens. Examples include (with number of fruiting specimens examined/percentage of ‘most inclusive’ EOO overlap; **Table S2**): *C. brevipes* (43/53%), *C. mannii* (282/83%), and *C. lebruniana* (70/29%). All these collections were of wild origin material. By comparison, the figures for wild coffee berry borer incidence are *C. canephora* [149/100% (30 coffee berry borer records)], *C. liberica* [147/95% (1 record)], and *C. mayombensis* [81/39% (1 record)]. Hence, we would expect, in the absence of other factors, for species such as *C. mannii* to show evidence of coffee berry borer infestation.

What is the likely host status of the other plant species surveyed (**Table 1**), i.e., on those plants assumed to be hosts (Beille, 1925), or believed to be hosts based on the coffee berry borer presence reports by (Schedl, 1960) as summarized by Vega et al. (2012)? There might be some doubt for the host status of those species and genera for which we sampled large numbers of species in fruit, e.g. *Pleiocarpa pycnantha* (Apocynaceae) (120 fruiting specimens), *Cathormion altissimum* (Leguminosae) (131 fruiting specimens), *Pycnanthus angolensis* (Myristicaceae) (111 fruiting specimens), and several Rubiaceae genera (five of which had more than 150 fruiting specimens). However, the number of fruiting specimens examined for these African plant groups (species and genera) is still low compared to the 2,725 fruiting specimens examined for African coffee (*Coffea*) species (**Table 1**). Further examination of herbarium collections and other museum material is warranted. We should also consider the coffee berry borer specimen (Musée du Congo, Lusambo, Jan. 1925) held at NHM, on which the collector records *Caesalpinia pulcherrima* as the host. Based on our findings, a species of *Hypothenemus* on *Mammea africana* that looks like *H. hampei* on initial identification with a binocular microscope, but turned out not to coffee berry borer after viewing with micro-CT for SEM, it is also possible that Schedl (Schedl, 1960) may have misidentified some specimens of *Hypothenemus*. Based on the high number of fruiting specimens examined for *Tricalysia* (Rubiaceae; 416 fruiting specimens) and *Oxyanthus* (Rubiaceae; 1143 fruiting specimens examined), with no evidence of coffee berry borer infestation, we have serious doubts for these genera as hosts.

Our herbarium survey and examination of museum collections of coffee berry borer suggest that in order to further investigate host status of this insect, dedicated field work in the wetter type of Guineo-Congolian forest (White, 1983), and particularly the Democratic Republic of Congo and western Uganda, would be warranted.

### Implications for Understanding the Evolution and Management of the Coffee Berry Borer

Our findings raise key questions on the evolution of host–plant acceptance, and more specifically, how the coffee berry borer became specialized on coffee seeds (*C. canephora*, *C. arabica*, *C. liberica*), a rare event considering that out of 181 described species in the genus *Hypothenemus*, only three feed on fruits/seeds (*H. hampei*, *H. eruditus*, and *H. obscurus*; Vega et al., 2015), and only one, the coffee berry borer, reproduces inside the coffee berry. As their common name implies, most bark beetles (Scolytinae) are wood boring beetles. Therefore, at some point, the coffee berry borer must have transitioned from a bark host in Africa to forest fruits (including coffee fruits). The transition to coffee fruits could have occurred within the forest, directly from bark to coffee fruits, or indirectly from bark to the fruits of non-coffee plants, and then to coffee fruits. It is possible, although seemingly unlikely given the time frame, that the transition from bark, or non-coffee fruit, could have happened simultaneously as coffee plantations became widely planted in tropical Africa. Whatever the pathway, survival on coffee has required a caffeine detoxification mechanism, likely involving the presence of caffeine-degrading bacteria in the alimentary canal (Ceja-Navarro et al., 2015), a factor complicating the recent shift from forest to cultivated plant. This is particularly important for survival on the seeds of *C. canephora*, which have a higher caffeine content than *C. arabica* (1.7% vs 1% on a dry weight basis, respectively; see Vega et al., 2015).

Knowing whether the insect infests other forest fruits would result in a better understanding of the evolution of coffee berry borer, and could result in a search for natural enemies of the insect in those habitats, which might be suitable for use as classical biological control agents elsewhere (Vega et al., 2012). We found no evidence of alternate hosts in our survey.

On the basis of high genetic diversity in sampled populations of the coffee berry borer from cultivated coffee in Java, Gauthier (2010) stated “It seems very likely that there are some indigenous but unsampled populations of the beetle at least in Java.”

We suggest that the coffee berry borer is unlikely to be indigenous to Java or Asia based on the few and sparsely distributed wild coffee species and the total lack of recorded and anecdotal wild occurrence records. The high genetic diversity of the insect in Java compared to rest of Asia is probably due to repeated introductions of the insect (via coffee plants, fruits, and possibly seeds only) from Africa to various research stations and research institutes in Java during the late 19^th^ century, and more particularly, during the early 20^th^ century, including material from the Congo (Democratic Republic of Congo, Republic of the Congo) and Uganda (Cramer, 1957).

### Herbarium Collections for Non-Traditional Uses

Stern and Eriksson (1996) present an argument for increased and non-traditional use of herbarium specimens if collected samples represented more of the conditions the plants face in nature. For example, they suggest that it would be useful to include diseased samples as well as material that exhibit insect-plant associations. Such material could be useful for plant pathologists, ecologists, and entomologists, respectively. In relation to insects, they write: “It is therefore unusual to find evidence of ecological associations in herbarium specimens, as most such samples derive from healthy, typical-looking individuals thoroughly cleaned during preparation. We suggest that the physical evidence of insect-plant associations be collected to a larger extent, to document these parasitic or mutualistic relationships.” Others further expand on this concept. For example, Veenstra (2012) concludes that an obvious limitation in the use of herbarium specimens for assessing the geographic distribution of an insect is that the lack of insect specimens does not necessarily imply they were not present in the site where the plants were collected. The reason might be that plant collectors might avoid less than perfect specimens, i.e., they might avoid specimens that show insect damage. Similarly, Abbott et al. (1999) state that evidence of leafminers in the herbarium samples they examined might be biased because “Botanical collectors usually select material to press and often avoid gathering blemished specimens.” It is also important to keep in mind whether the herbarium specimens present “seasonal collection bias” (*sensu*Abbott et al., 1999), which might preclude detecting an insect. It is important to note that if we start collecting specimens expressly to document insect associations but do not mention on the herbarium sheet label that this was the impetus, we risk biasing assessments of insect damage and how it has changed over time (Meineke et al., 2018).

Even though collecting plant specimens that show insect associations might be beneficial to plant pathologists, ecologists and entomologists, modern day botanical collectors are obviously focused on the plant material itself, as were their predecessors. Including associated organisms (e.g., insects associated with the plant) in the collected material could complicate transportation and customs inspection. Webber et al. (2007) conjectures that even though some *Ryparosa* herbarium specimens exhibit the traditional associations with ants (e.g., ant entry holes, swollen stem domatia), early collectors “often showed a bias against collecting colonized material because of the logistical complications caused by the associated ants.”

We examined 18,667 specimens in our survey, to find just 72 confirmed incidences, and only 36 wild records, for coffee berry borer hosts. This represents a substantial investment for what seems to be a low return of information. Nevertheless, we were able to (1) confirm literature and anecdotal reports indicating that the coffee berry borer is indigenous to tropical Africa; (2) confirm that *C. canephora* as an important host of the coffee berry borer; (3) show that the wetter type of Guineo-Congolian forest (White, 1983) is the preferred or exclusive habitat in Africa; and (4) conclude that certain plant genera, previously reported as hosts, are either not or are rare hosts for the coffee berry borer.

We have also established that micro-CT for SEM is extremely useful as a non-destructive sampling and visualization technique for cryptic insects on botanical specimens. Due to the fragile nature of herbarium specimens, the cryptic nature of insects within fruits, and the fact that destructive sampling is not an option, it is essential to have a reliable technique that allows visualizing minute insects in bored galleries within the fruits.

## Conclusions

Our herbarium survey confirms literature and anecdotal reports indicating that the coffee berry borer is indigenous to tropical Africa, and that coffee species, particularly *C. canephora*, are important hosts. We identify the wetter type of Guineo-Congolian forest (White, 1983) as either the preferred or exclusive native habitat of the coffee berry borer. Other than coffee, we find no evidence of other naturally occurring hosts, although do not rule out the possibility that they exist. The specificity of coffee berry borer entry/exit hole position, as identified here, and the presence of a caffeine detoxification mechanism, involving the presence of caffeine-degrading bacteria in the alimentary canal of the coffee berry borer (Ceja-Navarro et al., 2015), infers a certain degree of specificity between the insect and the coffee host.

## Data Availability Statement

The key datasets generated for this study are included in the manuscript/**Supplementary Files**; additional data can be requested from the corresponding authors.

## Author Contributions

FV and AD conceived and designed the experiments. LS, AD, ND, TG, RO’S, and FV conducted the experiments. AD, RO’S, and JM analyzed the data; FV, AD, and JM wrote the manuscript. All authors read and approved the manuscript.

## Funding

This research was funded by Specific Cooperative Agreement 58-1275-1-152F between the Sustainable Perennial Crops Laboratory (United States Department of Agriculture, Agricultural Research Service) and Royal Botanic Gardens, Kew.

## Conflict of Interest

The authors declare that the research was conducted in the absence of any commercial or financial relationships that could be construed as a potential conflict of interest.
