# Supplementary material for: Elucidation of Hosts, Native Distribution, and Habitat of the Coffee Berry Borer (Hypothenemus hampei) Using Herbaria and Other Museum Collections
Source: Front Plant Sci. 2019 Oct 1;10:1188. doi: 10.3389/fpls.2019.01188 (PMC6781851; doi:10.3389/fpls.2019.01188)
Supplement: Supplementary file 1 [file Table_1.docx]

**Supplementary Material**

**Elucidation of Hosts, Native Distribution, and Habitat of the Coffee Berry Borer (*Hypothenemus* *hampei*)**

**Using Herbaria and Other Museum Collections**

**Fernando E. Vega, Lucy T. Smith, Nina Davies, Justin Moat, Tomasz Góral, Robert O’Sullivan, and Aaron P. Davis**

**Table S1**. Coffee (*Coffea*) herbarium collections with incidence of coffee berry borer, with country of collection, year of collection (in Roman), collector’s name and collection number (in italics) and herbarium (in parentheses, abbreviations after Holmgren et al. 1990).

**Collections of cultivated origin**

***Coffea arabica*** Kenya. 1936, *McDonald H/3* (WAG); Democratic Republic of Congo (Zaire). 1912, *Chevalier 28367* (P).1912, Chevalier 28139 (P); 1925, *Robyns 912* (K); 1925, *Robyns 922* (K); 1925, *Robyns,895* (K). ***Coffea canephora*** Angola. 1930, *Gossweiler* *9434* (K). Benin. 1910, *Chevalier 23389* (K). Cameroon. 1918*, Annet 45* (P); 1995, *Demissew 5106* (WAG); 1945, *Aubréville 147* (P). Gabon. 1900, *Chalot 55* (P); Gabon, 1901, *Chalot s.n.* (K). Java. 1960, *Kuswata 16* (P). Sao Tome, 1993, *Figueiredo & Arriegas 76* (K). Uganda. 1913, Dummer 333 (K); 1921, *Snowden 742* (BM). Democratic Republic of Congo (Zaire). 1912, *Chevalier 28022* (P); 1931, *Corbisier-Baland 905* (P); 1912, *Chevalier 28363* (P); 1912, *Chevalier 28365* (P); 1923, *Goosens 4563* (BR); 1906, *Pynaert 390* (BR); 1903, *Laurent s.n. [b256]* (BR); 1903-1904, *Laurent s.n. [b257]* (BR); 1979, *Lejoly 5495* (BR); 1925, *Robyns 894* (BR); 1925, *Robyns 923* (BR); 1906, *Pynaert 671* [b393] (BR). **Coffea congensis** Ivory Coast. 1983*, Zadi Koubi 402* (K). Democratic Republic of Congo (Zaire). 1912, *Chevalier 28370* (P); ***Coffea* x *crameri*** Java. 1914, *Chevalier 10* (P). ***Coffea liberica*** Ivory Coast, 1928, *Court B34030* (P). Democratic Republic of Congo (Zaire). 1975, *Lisowski 41062* (BR); 1909, *Seret s.n. [b745]* (BR); 1909, *Robyns 908* (BR); 1909, *Seret b683* (BR). ***Coffea stenophylla*** Ivory Coast. 1984, *Hepper & Maley* 7728 (K).

**Collections of wild origin**

***Coffea canephora*** Angola. 1909, *Gossweiler 4768* (K). Cameroon. 1987, *Manning 1631* (K). Ivory Coast, 1963, *deWilde 156* (WAG). South Sudan, 1980, *Vollesen & Friis* 491 (BR). Uganda. 1941, *Thomas 4027* (K); 1926*, Maitland 1368* (K); 1932*, Hazel 155* (K); 1933, *Thomas 1032* (K); 1952, *Thomas 751* (K); 1935, *Taylor 3328* (BM). Democratic Republic of Congo (Zaire). 1934, Soors s.n. [b177] (BR); 1934, *Soors s.n. [b178]* (BR); 1938, *Myers 10212* (K); 1935, *Louis 882* (K); 1938, *Myers 10180* (K); 1938, *Myers 10212* (K); c. 1902, *Gillet b279* (BR); 1924, *Goosens 4766* (BR); 1959, *Chambon 10* (BR); 1936, *Gillardin 177* (BR); 1959, *Goffinet 55* (BR); 1935, *Louis 410* (BR); 1935, *Louis 838* (BR); 1902*, Gentil s.n. [b307*] (BR); 1902, *Gentil,96 (b191)* (BR); 1935, *Louis 882* (BR); 1936, *Louis 2994* (BR); 1935, *Louis 882* (WAG); 1959, *Chambon 10* (WAG); 1957, *Liben 3250* (WAG). ***Coffea liberica*** Uganda 1932, *Hazel 154* (K). *Coffea mayombensis* Nigeria. 1946, *Keay & Onochie 21726* (K).

**Cultivated/wild origin unknown**

***Coffea canephora*** African country unknown, 1917, *De Wildem 472/65* (P). Democratic Republic of Congo (Zaire). 1906, *Pynaert s.n.[b292]* (BR).


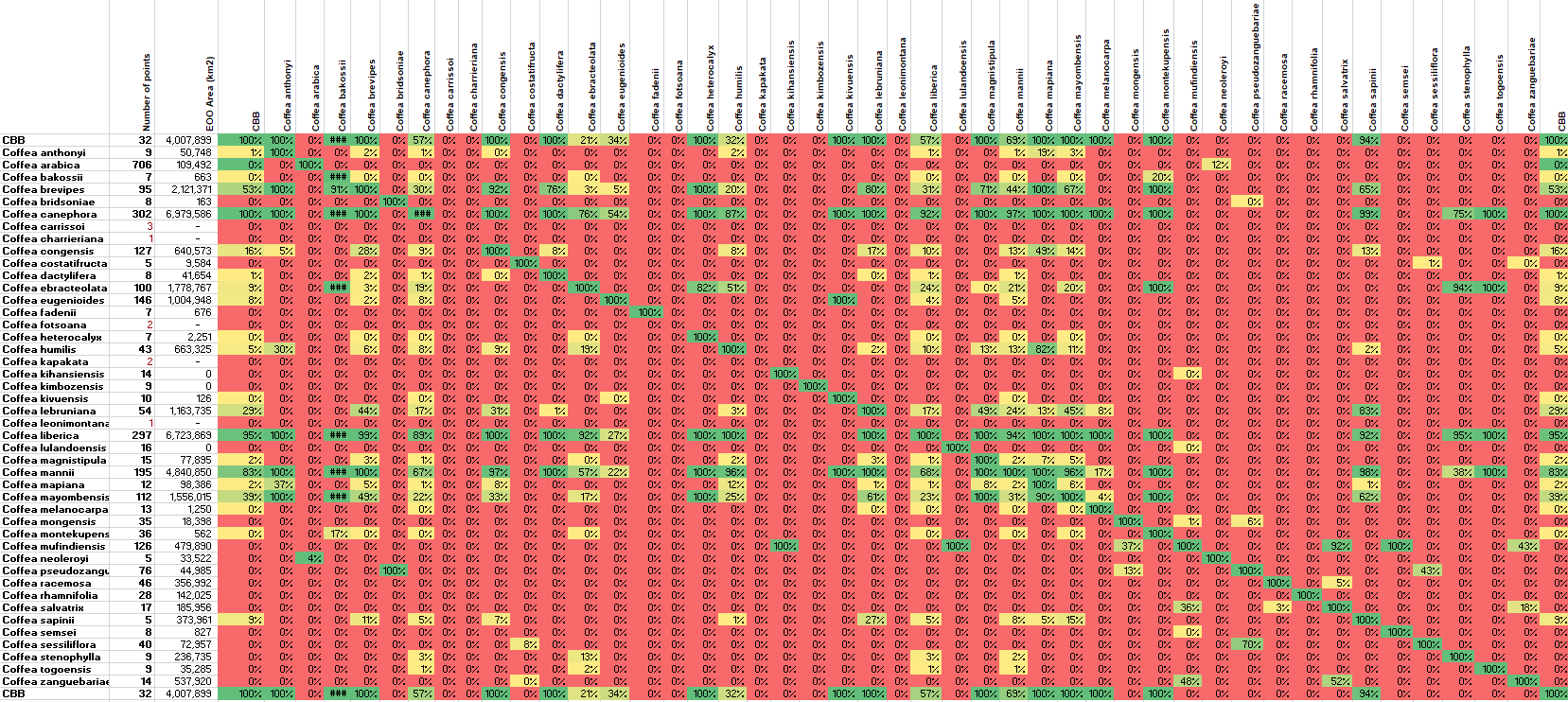


**Table S2.** Extent of Occurrence (EOO) overlap for CBB (based on the results of this study) and wild coffee species, with number of points, and EOO. Most inclusive [% coffee species EOO overlap with CBB EOO; first and last column (vertical axis). Least inclusive distribution [% CBB EOO overlap with coffee species EOO; first and last row (horizontal axis ).
